# Application of antisense oligonucleotide drugs in amyotrophic lateral sclerosis and Huntington’s disease

**DOI:** 10.1186/s40035-025-00466-9

**Published:** 2025-01-21

**Authors:** Kaili Ou, Qingqing Jia, Dandan Li, Shihua Li, Xiao-Jiang Li, Peng Yin

**Affiliations:** State Key Laboratory of Bioactive Molecules and Druggability Assessment, Guangdong Key Laboratory of Non-Human Primate Research, Key Laboratory of CNS Regeneration (Ministry of Education), Guangdong-Hongkong-Macau Institute of CNS Regeneration, Jinan University, Guangzhou, 510632 China

**Keywords:** Amyotrophic lateral sclerosis, Huntington’s disease, Clinical trial, Antisense oligonucleotide

## Abstract

Amyotrophic lateral sclerosis (ALS) and Huntington’s disease (HD) are diverse in clinical presentation and are caused by complex and multiple factors, including genetic mutations and environmental factors. Numerous therapeutic approaches have been developed based on the genetic causes and potential mechanisms of ALS and HD. Currently, available treatments for various neurodegenerative diseases can alleviate symptoms but do not provide a definitive cure. Gene therapy, which aims to modify or express specific proteins for neuroprotection or correction, is considered a powerful tool in managing neurodegenerative conditions. To date, antisense oligonucleotide (ASO) drugs targeting the pathological genes associated with ALS and HD have shown promising results in numerous animal studies and several clinical trials. This review provides a comprehensive overview of the development, mechanisms of action, limitations, and clinical applications of ASO drugs in neurodegenerative diseases, with a specific focus on ALS and HD therapeutic strategies.

## Introduction

Neurodegenerative diseases are complex and multifactorial conditions encompassing a range of disorders, each characterized by unique pathological patterns, clinical manifestations, and underlying causes. These diseases are significant causes of mortality and disability, and pose substantial burdens on families and societies. Various strategies have been developed to address neurodegenerative diseases, including protein-based therapies, gene therapies, and stem cell therapies. However, these current treatments can only alleviate symptoms, but not provide a definitive cure for these conditions. In addition, antibody therapies only target specific aggregates such as amyloid-beta (Aβ) and microtubule-associated protein Tau. Although lecanamab (Biogen) [1] and donanemab (Eli Lilly) [2, 3], which were approved by FDA in 2023 and 2024 respectively, are in the clinical treatment of AD patients, some antibody drugs, including crenezumab, bapineuzumab, solanezumab, gantenerumab, and aducanumab have already been discontinued for clinical application. Gene therapies that aim to confer neuroprotection or correction by modifying or expressing target genes, are a promising tool for treating neurodegenerative diseases. Additionally, major challenges remain in the application of gene therapy, such as finding out the precise delivery of genes in the central nervous system (CNS) and achieving a certain transduction efficiency [4].

Nucleic acid drugs, mainly including mRNA drugs and small nucleic acid drugs, play a crucial role in gene therapy. The mRNA drugs, including mRNA vaccines, enter the cytoplasm to translate specific proteins or antigens. When these substances are intra- or extra-cellular, they stimulate the immune system to produce immune responses. The small nucleic acid drugs consist of short chains of nucleotides designed to interfere with the expression of target genes and achieve therapeutic purposes [5], such as antisense oligonucleotides (ASOs) [6], small-interfering RNA (siRNA) [7], microRNA (miRNA) [8], small activating RNA (saRNA), and RNA aptamers [9]. ASOs are functional molecules that specifically target and modify RNA transcripts to slow down or halt the progression of rare genetic diseases. In detail, ASOs are artificially synthesized small-sized, single-stranded nucleic acids, typically 13–30 nucleotides in length. ASOs are advantageous over siRNAs in terms of targeting both nuclear and cytoplasmic RNAs. Upon entering cells, they bind to complementary mRNA based on the principle of base pairing, to silence the gene. By reducing target RNA transcript levels, ASOs lead to limited expression of toxic proteins or alter transcript sequences through splice-modulation to restore protein function [6]. In the following, we will focus on the development history, mechanisms of action, and clinical applications of ASO drugs in neurodegenerative diseases, especially amyotrophic lateral sclerosis (ALS) and Huntington’s disease (HD).

## Clinical development of ASOs

### First-generation ASOs

In the first-generation ASOs, backbone modification is used, including replacing the hydrogen atom of the phosphodiester bond with sulfur (thioate), methyl (methylphosphonate), or amino (amidate) groups [10]. The most widely used backbone modification is phosphorothioate (PS), where the non-bridging hydrogen atoms of the phosphodiester bond are replaced by sulfur atoms. This modification reduces the hydrophilicity of the nucleotide and enhances resistance to nucleases [11]. While the PS backbone modification enhances protein binding, facilitates cellular uptake, and improves tissue distribution [11, 12], it can also interfere with the function of normal proteins and activate the complement system, leading to inflammatory reactions [13] . Fomivirsen (vitravene), the first approved ASO drug in 1998, was withdrawn from the market in 2002 in Europe and in 2006 in US by Novartis [14]. Fomivirsen is a 21-nucleotide PS oligonucleotide used to treat cytomegalovirus (CMV) infection. Fomivirsen contains a sulfur modification to extend its half-life. It specifically binds to the mRNA of CMV-IE2 (cytomegalovirus immediate-early 2) and is recognized by RNaseH, leading to the hydrolysis of the target mRNA and inhibition of IE2 protein synthesis, thereby suppressing CMV proliferation and achieving therapeutic effects [15]. PS-modified ASOs are not completely protected from nucleases, which can reduce their hybridization to target mRNAs. Also, these ASOs can interact with proteins, which may lead to negative side effects [16]. Based on these, the first-generation ASOs have largely been abandoned in pipelines and have not been used as therapeutics for neurodegenerative diseases.

### Second-generation ASOs

Second-generation ASOs are optimized to enhance specificity, stability, biostability, and safety compared to the first-generation, aiming to more effectively treat various diseases [17–19]. In second-generation oligonucleotides, nucleotides are modified at the 2’ position of the ribose sugar. The hydroxyl group (-OH) at the 2’ end of the ribose can be replaced by various substituents such as MOE, OMe, or F. Among them, 2’-MOE is the most used modification in ASO drugs. Each change at the 2’ position improves the nuclease resistance, half-life, and target affinity [20]. The first FDA-approved antisense nucleic acid drug based on 2’-MOE is kynamro (mipomersen) approved in 2013 for treating familial hypercholesterolemia [14], which was, however, withdrawn in 2019. Kynamro (mipomersen) is a fully PS-modified, 20-nucleotide ASO gapmer, containing a central 10-base DNA and two 2’-O-MOE-modified nucleotide segments (5-nucleotide ‘wings’) at the 5’ and 3’ ends, respectively [21]. Tegsedi (Inotersen) approved in 2018, also a fully PS-modified ASO gapmer with MOE wings and a central DNA, is used to treat hereditary transthyretin amyloidosis with polyneuropathy [22, 23]. On the other hand, spinraza (nusinersen), the first drug approved by FDA for spinal muscular atrophy (SMA) in 2016, functions in a completely different mechanism. This fully PS/MOE-modified 18-nucleotide RNA is used to treat SMA and modulates the alternative splicing of the survival motor neuron 2 (*SMN2*) gene [24]. Another MOE-based ASO gapmer, waylivra (volanesorsen), was approved by the European Medicines Agency (EMA) in 2019 as an adjunctive therapy for adult patients with familial chylomicronemia syndrome [25].

### Third-generation ASOs

Third-generation ASOs offer significant improvements in specificity, stability, cellular uptake and efficacy with reduced immunogenicity, making them a promising choice for therapeutic intervention [26, 27]. The third-generation ASOs including peptide nucleic acids (PNAs) and phosphorodiamidate morpholino oligomers (PMOs), incorporate modifications of ribose with alternative chemistries. PNAs are characterized by the substitution of the sugar-phosphate backbone with a pseudopeptide polymer, incorporating N-2-(aminoethyl)-glycine units as chemical modifications. Due to their neutral and non-natural backbone, PNAs exhibit resistance to enzymatic degradation compared to the unmodified ASOs and demonstrate strong binding affinity to RNA sequences [28]. PMOs are morpholine-based, short single-stranded DNA analogs in which the ribose sugar is replaced by a morpholine ring, and the phosphodiester bond is substituted by phosphorodiamidate linkages. The morpholine ring enhances water solubility, and the absence of a carbonyl group prevents protease and esterase cleavage [29]. Eteplirsen (EXONDYS 51) and golodirsen (VYONDYS 53), approved by FDA in 2016 and 2019 respectively for treatment of Duchenne Muscular Dystrophy (DMD), are both morpholino oligomer analogs. They specifically bind to exon 51 and exon 53 of the dystrophin pre-mRNA, resulting in the removal of the corresponding exons and production of truncated dystrophin protein [30, 31]. Viltepso (viltolarsen) approved in 2020, is also a third-generation ASO developed for the treatment of DMD by promoting exon skipping [32]. Clinical trials have shown that viltolarsen can increase dystrophin production and improve muscle function in patients with DMD [33, 34] (Table 1).

**Table 1:** Chemical modifications and development of ASOs

Chemical modification	Modification type	Chemical construction	Advantage	Disadvantage	Representative drug/Sequence
First-generation backbone modification	PS		Increases stability Improves distribution tissues	Slightly reduces binding affinity compared to phosphodiester Causes immunse response / cytotoxicity at high concentrations	Fomivirsen 5’-GCGTTTGCTCTTCTCTTCTTGCG-3’
Second-generation ribose modification	2’-O-Me		Increases stability Improves potency Reduces non-specific toxicities	Lower affinity than most other modifications Results in inefficient cleavage of the target RNA by RNase H	Mipomersen 5’-GCCTCCAGTCTGCTTCGCACC-3’ Inotersen 5’-TCTTGGTTACATGAAATCCC-3’ Volanesorsen 5’-TATTTCGACCTGTTCTTCGA-3’
	2’-MOE				
	2’-F				
Third-generation alternative chemistries	PNA		Improves binding affinity Excellent nuclease resistance Does not support RNase H	Potential liver toxicity and low delivery efficiency Rapid clearance Poor uptake/pharmacokinetic properties	Eteplirsen 5’-CTCCAACATCAAGGAAGATGGCATTTCT-3’ Golodirsen 5’-GTTGCCTCCGGTTCTGAAGGTGTTC-3’ Vitolarsen 5’-CCTCCGGTTCTGAAGGTGTTC-3’
	PMO				

## Mechanisms of actions of ASOs

**Fig. 1:**
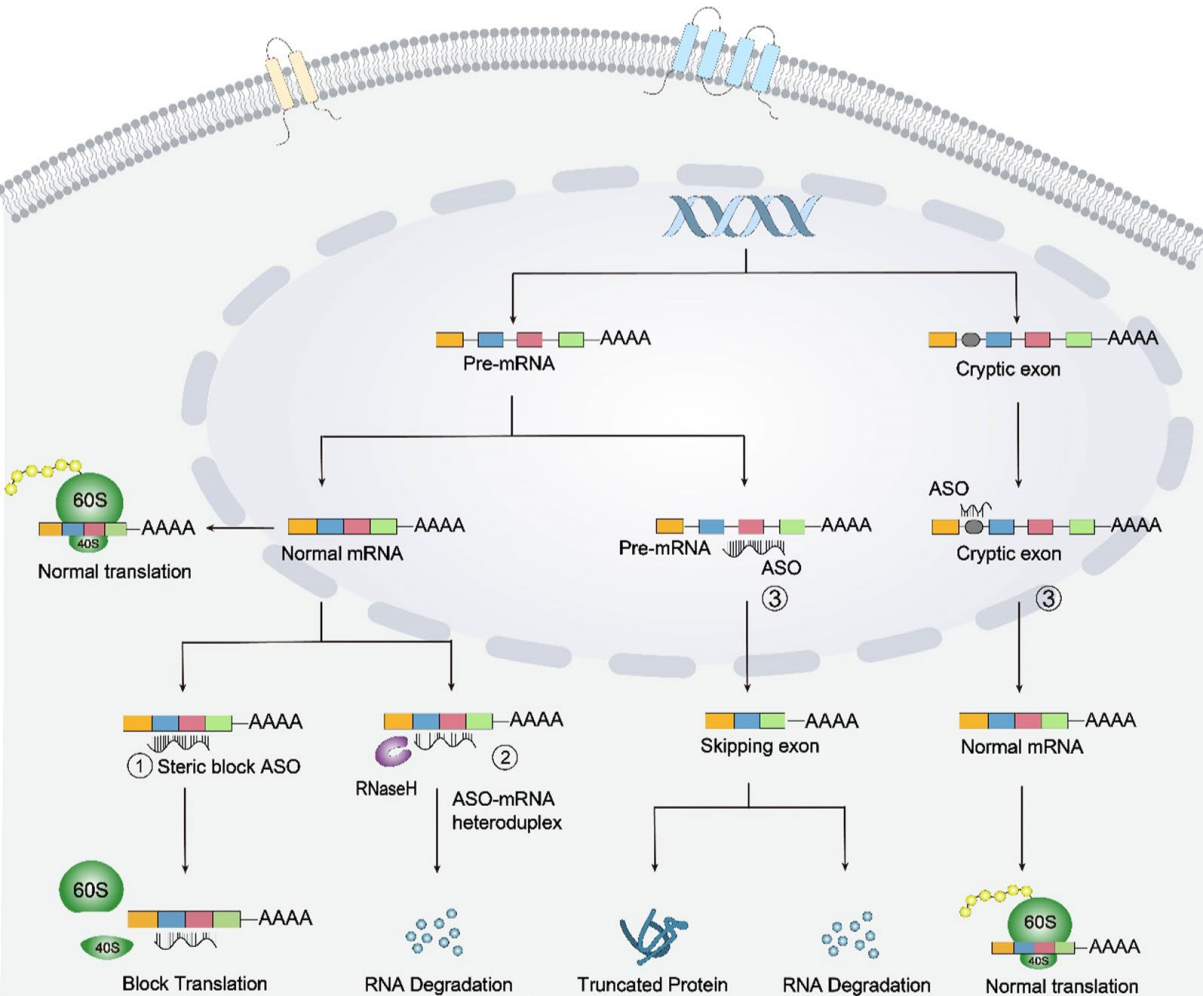
Mechanisms and comparison of ASOs. The ASOs regulate RNA function through different mechanisms. ① Steric blocking. The ASOs prevent ribosome from binding with mRNA, blocking translation. ② Activating RNase. The ASOs bind to target mRNA through base pairing and recruit ribonuclease H1 (RNase H1), leading to degradation of the target mRNA. ③ Splicing modulation. When binding to an exon of pre-mRNA, the ASOs cause exon skipping or inclusion, resulting in production of a truncated protein or pre-mRNA degradation. When binding to the cryptic splice sites in introns, ASOs can inhibit abnormal splicing, thereby increasing the expression of normal gene

ASOs can modulate gene expression mainly through three approaches based on their chemical properties and target sites [35]. The first approach involves steric hindrance upon ASO binding to mRNAs, thereby preventing the mRNAs from entering the ribosome for protein translation, resulting in downregulation of gene expression [36]. The second approach is that ASOs bind to target mRNAs through base pairing and recruit ribonuclease H1 (RNase H1) to cleave the target mRNAs. The third approach involves splicing modulation. When binding to an exon of a pre-mRNA, ASOs can alter the splicing patterns via exon skipping or inclusion, which are the most common alternative splicing types. For instance, *SMN2* gene, related to SMA disease, often produces the short and unstable proteins due to the skipping of exon 7 [37]. In DMD patients, the deletions involving exons 45 and 55 can lead to the production of incorrect dystrophin protein [38]. The exon-inclusion ASO drug spinraza for SMA, and the exon-skipping drugs EXONDYS51 and VYONDYS53 for DMD have been approved [14]. The modified mRNAs after exon excision leads to production of a truncated protein, or is degraded through the nonsense-mediated mRNA decay pathway [37, 39, 40]. When binding to the cryptic splice sites in introns, ASOs can inhibit abnormal splicing, thereby increasing the expression of the wild-type (WT) gene via normal splicing [41, 42]. ASOs can also target the 3’ poly(A) tail of pre-mRNA in the 3’ untranslated region (UTR) [43] or the cap site within 5’UTR to alter protein synthesis and translation [44–47] (Fig. 1).

## Application of ASOs in ALS

ALS is a neurodegenerative disease characterized by selective degeneration of motor neurons, leading to progressive muscle weakness and ultimately respiratory failure. Most ALS patients die within 2 to 5 years after the onset of symptoms [48]. About 10% of ALS patients are familial ALS, and so far, more than 30 ALS-related genes have been identified in familial ALS cases [48, 49]. However, the remaining 90% of cases are clearly sporadic ALS [50]. Pathogenic variants in superoxide dismutase 1 (*SOD1*), TAR DNA binding protein (*TARDBP*), fused in sarcoma (*FUS*) and chromosome 9 open reading frame 72 (*C9ORF72*), the most common ALS-associated genes, account for approximately 60% of familial cases and about 10% of sporadic ALS. The available treatments for ALS show limited efficacy in slowing disease process [51, 52]. To date, four drugs have been approved by FDA for ALS, including riluzole approved in 1995 [53], edaravone approved in 2017 [52], relyvrio approved in 2022 [54] and tofersen approved in 2023 [55]. Rilutek (riluzole) is a small molecule that modulates voltage-dependent Na^+^ channels to mitigate motor neuron excitotoxicity. Radicava (edaravone) is a small-molecule antioxidant. AMX0035 (relyvrio) alleviates endoplasmic reticulum stress and mitochondrial dysfunction. However, it has been withdrawn by Amylyx recently, as it did not meet the primary end point of change in ALS Functional Rating Scale-Revised (ALSFRS-R) in Phase III PHOENIX trial of 2024 [54, 56]. As a result, for greater efficacy and longer-lasting effects, molecular therapeutic approaches are gaining increasing attention in ALS, focusing on inhibiting the toxic gain-of-function (GOF) of ALS-related genes *SOD1*, *C9ORF72*, *FUS* and *ATXN2*.

### ASOs targeting the *SOD1* gene

*SOD1* was the first gene identified in 1993 to be associated with ALS. *SOD1* encodes a superoxide dismutase enzyme that plays a crucial role in antioxidant defense [57]. The exact mechanism by which *SOD1* mutations lead to ALS is not fully understood. It is hypothesized that the toxicity is related to the aggregation of mutant SOD1 protein, suggesting a GOF mechanism [58].

The first ASO therapy targeting *SOD1* mRNA via RNase H1 is the ISIS 333611, a 2′-MOE gapmer, granted orphan drug status by FDA in 2007 [59]. Animal studies showed that this ASO led to a dose-dependent reduction in SOD1 mRNA and protein levels in human SOD1^G93A^ transgenic rats, resulting in 40%−60% decreases of SOD1 in the brainstem and spinal cord [59]. The lowering of SOD1 expression was well tolerated, leading to a delay in disease onset and a 37% extension of survival after disease onset in the transgenic rats. Similar effects were achieved by infusing the ASO into the right lateral ventricle of non-human primates, such as rhesus monkeys, for two weeks [59]. These promising preclinical results prompted the initiation of a Phase I, double-blind, placebo-controlled clinical trial (NCT01041222) starting in 2010, to assess the safety and tolerability of intrathecally administered ASO 333611 in *SOD1* ALS patients [60]. This trial marked the first instance of an experimental antisense drug being intrathecally administered to patients for treatment. A single course of a low dose (0.15 to 3 mg) was delivered over 12 h through slow intrathecal infusion. Despite the absence of observable reductions in SOD1 protein levels in the cerebrospinal fluid (CSF), this trial was pivotal for ASO therapy, showcasing the safety and efficacy of intrathecal ASO infusion in humans. It also established a framework to improve the progression of ASOs from initial selection to clinical trials [60, 61].

BIIB067, also qalsody (tofersen), is a second-generation ASO that targets different regions of *SOD1* pre-mRNA with a higher potency to suppress SOD1 mRNA expression than ASO 333611 [62]. In the early stages of preclinical experiments, the ASOs were delivered intraventricularly or intrathecally to the CSF of mice and rats expressing human SOD1^G93A^. The ASOs were shown to be distributed throughout the entire brain and spinal cord, leading to a dose-dependent reduction in human *SOD1* mRNA, and significantly delayed disease onset and prolonged survival of SOD1^G93A^ mice and rats. Intrathecal injection of the ASOs in cynomolgus monkeys also resulted in a widespread dose-dependent reduction of SOD1 mRNA in the CNS [62]. These findings laid foundation for human clinical trials in SOD1 ALS patients. In a Phase I/II clinical trial in 2020 (VALOR; NCT02623699), subjects received intrathecal injections of the ASO or placebo for 12 weeks. No serious adverse events were reported, and a dose-dependent reduction in SOD1 protein concentration was observed in the CSF of both low-dose and high-dose tofersen groups [63]. Unfortunately, in a Phase III clinical trial (NCT02623699), which started in 2016, there was no significant change in the clinical endpoints of patients, including the ALSFRS-R, motor function, and pulmonary function after 28 weeks of treatment [64]. After the Phase III trial, it was also transitioned to a non-randomized open-label extension trial in 2017 (NCT03070119), and the results at 52 weeks showed a slower decline in respiratory function and muscle strength in patients [65]. Based on the observed reduction in plasma neurofilament light chain (NfL) levels in patients receiving treatment, in 2023, tofersen received the FDA’s accelerated approval for the treatment of ALS patients with *SOD1* gene mutations.

### ASOs targeting the *C9ORF72* gene

*C9ORF72* is the most common genetic cause of both familial and sporadic ALS, accounting for approximately 40% of familial cases and 5% of sporadic cases. It is also the most common genetic cause of frontotemporal dementia, representing about 25% of cases. *C9ORF72* mutations involve a repeat expansion of the hexanucleotide GGGGCC in the first intron. WT alleles typically contain fewer than 30 repeats, while pathogenic alleles have been documented to contain 700–1600 repeats [66]. *C9ORF72* generates three different transcript variants: variants 1 (V1), V2, and V3 [67–69]. Although the underlying pathogenic mechanism of the G4C2 repeat expansion is largely unknown, one potential pathogenic mechanism involves the production of abnormal dipeptide repeat (DPR) proteins through repeat-associated non-AUG translation [70]. Another potential pathogenic mechanism is the RNA-mediated toxicity, where the formation of RNA foci and sequestration of specific RNA-binding proteins are linked to the misregulation of RNA processing in repeat expansion diseases [71].

To minimize the impact of ASOs on the normal function of C9ORF72, *C9ORF72*-targeting ASO BIIB078 (formerly IONIS-C9_Rx_) was pursued. Jiang et al*.* injected ASOs targeting the G4C2 repeat expansion in the C9 mice, observing a sustained reduction in RNA foci and DPRs in the cortex and spinal cord, along with improvements in cognitive impairments [72]. Furthermore, in a single patient harboring the mutant *C9ORF72* gene, CSF levels of poly-glycine-proline (poly-GP) DPRs, a stable biomarker of *C9ORF72*-ALS, were decreased following multiple intrathecal administrations of ASOs [73, 74]. In 2018, Biogen began the Phase I trial of BIIB078 in ALS adults with *C9ORF72* expansions. The initial clinical trial aimed to evaluate the safety and tolerability of the ASO with a PS backbone, which selectively targets *C9ORF72* transcript V1 and V3 carrying the expansion that leads to RNase H degradation. This trial (NCT03626012) was a randomized, placebo-controlled study involving 106 participants with *C9ORF72*-ALS (excluding fast progressors) who received escalating doses of BIIB078 or a placebo via intrathecal infusion. Although BIIB078 was well tolerated, it did not achieve any of the predefined endpoints related to the changes in ALSFRS-R, slow vital capacity, and muscle strength. Therefore, development of this therapy was discontinued in 2022.

The stereopure oligonucleotide WVE-004, has shown promising results in preclinical assessment for treatment of *C9ORF72*-associated ALS or frontotemporal dementia (FTD) [75]. Injection of the ASO WVE-004 into the brain ventricles of *C9ORF72* BAC transgenic mice significantly reduced V3 transcripts and poly-GP protein levels, and these reductions lasted for at least 6 months without disrupting the total expression of C9orf72 protein [76]. A Phase I/II clinical trial (NCT04931862) has been launched to assess the safety, tolerability, and effectiveness of this variant-selective, stereopure design and a phosphoryl guanidine backbone-based ASO WVE-004 in 2021 [76]. However, in individuals with ALS or FTD caused by *C9ORF72* expansion, WVE-004 did not demonstrate clinical advantages over a 24-week period, which targeting a region close to the exon 1b-intron junction. This prompted halting of additional advancements in 2023. It should be noted that the CSF level of poly-GP showed a notable 48% decrease, but this reduction did not correlate with the stabilization or enhancement of functional outcomes compared to the placebo group [76].

Recently, a novel chemically modified ASO called afinersen has been designed to target the intronic region surrounding the GGGGCC repeat expansion. It works by inhibiting the expression of V1 and V3 variants while permitting basal levels of V2. Studies in large animals such as sheep and non-human primates showed that afinersen is non-toxic to large animals and has similar effects as in mice [74]. Based on this, with authorization from the FDA, afinersen was tested for the treatment of a 60-year-old patient with *C9ORF72*-related ALS (IND141673). It demonstrated extensive distribution within the CSF, leading to an 80% reduction in CSF level of poly-GP compared to baseline, signifying its efficacy in mitigating the consequences of *C9ORF72* expansion. Notably, the patient’s ALSFRS-R remained stable throughout the treatment course. Currently, this ASO is undergoing a Phase I clinical trial [74, 77].

### ASOs targeting the *FUS* gene

*FUS* mutations represent 4% of familial ALS cases, and are linked to a severe and early-onset variant of ALS [78]. *FUS* encodes an RNA-binding protein that is primarily involved in DNA repair and RNA metabolism [79]. *FUS* mutations cause mislocalization of the FUS protein from the nucleus to the cytoplasm, resulting in the formation of cytoplasmic inclusions composed of the abnormally-modified FUS proteins, as well as the interacted RNAs and other proteins [80, 81].

In preclinical studies, the ASO ION363 (jacifusen) targeting the sixth intron of *FUS* was injected into the lateral ventricle of FUS^P525L^ mice, resulting in reduced levels of mutant and WT FUS proteins in the mouse brain and spinal cord and delayed motor neuron degeneration[82]. The utilization of ION363 was prompted to application to a patient carrying the FUS P525L mutation after submitting a ‘compassionate use’ Investigational New Drug (IND) application to the FDA in 2022 [82]. Throughout the treatment period, the patient did not encounter any severe adverse event. However, the patient died from complications of the disease. Subsequent neuropathological analysis revealed that ION363 effectively diminished both WT and mutant FUS protein levels, resulting in a reduction of FUS aggregates. Notably, minimal nuclear FUS staining was observed in the spinal cord and motor cortex, and there was a decrease in FUS-containing aggregates within motor neurons compared to the untreated ALS-FUS P525L control, where FUS aggregates were prevalent [82].

The clinical effectiveness, safety, and pharmacological profile of ION363 (jacifusen) are currently under scrutiny in a Phase III clinical trial started from 2021 by Ionis, involving ALS patients with *FUS* mutations (FUSION; NCT04768972). The Phase III trial of jacifusen is a global multicenter study involving 64 participants. In the initial Phase of the trial, participants will be randomly assigned to receive either jacifusen or a placebo for 29 weeks. The subsequent phase of the trial will be an open-label period during which all participants will receive treatment with jacifusen for 73 weeks. Individuals demonstrating substantial functional deterioration in Part 1 will be transitioned to the open-label extension (Part 2) of the study. The primary endpoints involve the assessment of ALSFRS-R, time to rescue, and survival duration without ventilator assistance. Secondary endpoints encompass the evaluation of muscle and lung function, survival rates, and alterations in CSF FUS protein levels and neurofilaments. The results of this study are anticipated to be disclosed in 2025 [83].

### ASOs targeting the *ATXN2* gene

Transactivation response DNA-binding protein 43 (TDP-43) is a protein encoded by the *TARDBP* gene. TDP-43 is primarily expressed in the cell nucleus and plays multiple roles in physiological conditions, including pre-mRNA splicing, mRNA transport, and regulation of mRNA stability [84, 85]. Approximately 97% of ALS cases and 45% of FTD cases exhibit cytoplasmic inclusions containing TDP-43 in neurons. Abnormal TDP-43 protein aggregation and mislocalization in the cytoplasm is a significant pathological hallmark of ALS [86, 87]. However, there has been no application of ASO drugs targeting TDP-43 directly.

The trinucleotide repeat expansion in ataxin-2 (*ATXN2*) gene is a risk factor for ALS, which is associated with TDP-43 aggregation by regulating stress granule formation [88, 89]. Knockdown of *ATXN2* can delay disease progression and prolong survival of TDP-43 ALS mice [90]. ASOs targeting *ATXN2* can reverse the cytoplasmic mislocalization of nuclear proteins. An animal study also demonstrated that a single intravenous injection of *Atxn2* ASO reduced *Atxn2* mRNA level, extended survival and improved gait of TDP-43^Tg^/^Tg^ Atxn2^+/+^ mice [91–93]. In 2020, a randomized, placebo-controlled Phase I clinical trial (NCT04494256) was started to evaluate the safety and pharmacokinetics of intrathecal delivery of ASO BIIB105 (ION541). In 2022, it transitioned to a Phase I/II trial with increased enrollment of 98 participants and expanded efficacy endpoints, including changes in plasma biomarkers such as neurofilament light chain. Although BIIB105 significantly reduced CSF ataxin-2 protein levels, it did not affect plasma level of neurofilament light chain and clinical measures of function, such as breathing and strength. Consequently, Biogen and Ionis decided to discontinue the development of BIIB105 in 2024.

## Application of ASOs in HD

HD is an autosomal-dominant inherited neurodegenerative disorder caused by abnormal expansion of CAG repeat sequences in the huntingtin (*HTT*) gene on chromosome 4, which encodes a polyglutamine tract at the N-terminus of the protein. HD primarily affects the striatum and cerebral cortex, leading to a triad of motor disturbances, psychiatric abnormalities, and dementia [94, 95]. The pathogenesis of HD is believed to be mainly driven by the GOF of mutant HTT (mHTT) protein [96]. Therefore, reducing mHTT protein and controlling or eliminating its cellular toxicity are the main goals of HD gene therapy. FDA-approved drugs currently used for the treatment of HD-related chorea include tetrabenazine and deutetrabenazine. Tetrabenazine, approved in 2008, is an inhibitor of synaptic vesicular monoamine transporter 2 (VMAT2). It can decrease concentrations of dopamine, serotonin, norepinephrine, and other monoamines in the synaptic cleft by inhibiting VMAT2, thereby exerting an anti-chorea effect [97, 98]. However, this drug can cause neuroleptic malignant syndrome, exacerbate depression, and increase the risk of suicide. Deutetrabenazine, approved by FDA in 2017, is also a VMAT2 inhibitor, reducing the concentrations of monoamines in the synaptic cleft, and reducing presynaptic neuronal reuptake, with a longer half-life in plasma and fewer side effects [99–101].

Tominersen is an ASO drug that can target the CAG repeat sequence on the RNA strand produced by both normal and mutant *HTT* gene and mediate its degradation by the enzyme RNase H1. Participants in a Phase I/IIa study in 2018 were offered to take part in the open-label extension (NCT03342053) clinical trial with tominersen, where they received 120 mg of the ASO drug every 4 or 8 weeks during a period of 15 months. Preliminary results from the open-label extension study showed increases in CSF NFL concentrations on day 141, which decreased thereafter despite continued dosing with tominersen [102]. Based on these promising Phase I/IIa data, a large multinational Phase III study, GENERATION HD1 (NCT03761849), was started in 2019. The study was initiated with intrathecal dosing of tominersen every 4 or 8 weeks. However, dosing in this trial was halted based on the Independent Data Monitoring Committee’s recommendation in 2021. Although there were persistent dose-dependent decreases in CSF mHTT, the group receiving tominersen every 8 weeks performed worse on clinical rating scales and had higher frequencies of serious adverse events compared to placebo [103].

WVE-120101 and WVE-120102 are stereopure ASOs under investigation that target the expanded CAG repeat sequence in the *HTT* gene linked to two allelic variants of the HTT transcript [104]. A Phase I/IIa study (NCT02519036) in 2015 demonstrated that bolus intrathecal administrations of tominersen every 4 weeks at ascending doses from 10 to 120 mg induced ~ 40% reduction of mHTT in CSF with no serious adverse events after 4 doses [102]. PRECISION-HD1 (NCT03225833) and PRECISION-HD2 (NCT03225846) are two ASOs being tested against the expanded HTT protein, both started in 2017. While these drugs were found to be safe, they did not lower HTT protein levels as expected, leading to the discontinuation of the studies. The CAG repeat expansion in the *HTT* gene is associated with different single-nucleotide polymorphisms (SNPs) enriched in the mutant allele. SNP3 is present in the expanded alleles of approximately 40% HD adults [105]. SNP3 is an allelic variant associated with the expanded CAG repeat sequence in the HTT mRNA. WVE-003 is the only allele-selective HD candidate drug in clinical development. WVE-003 utilizes a modified phosphoramidate backbone that can increase tissue exposure, CNS half-life, and ASO potency [105]. WVE-003 preferentially reduces mHTT by targeting SNP3 present on the mHTT allele but absent on the WT HTT allele. In preclinical experiments, WVE-003 was injected into the lateral ventricle of HD mouse models (BACHD, YAC128, and R6/2 mice) and non-human primates and demonstrated inhibition of huntingtin protein and promotion of survival in HD model mice and non-human primates [106]. A Phase Ib/IIa multicenter, randomized, double-blind, placebo-controlled study (NCT05032196) was started in 2021 to evaluate the safety and tolerability of WVE-003 in adult patients with early-manifest HD carrying the targeted SNP3 (Table 2).

**Table 2:** Applications of ASOs in ALS and HD

Disease	ASO drugs	Modification type	Target	Animal model	Administration	Development stage	Manufacturer	Clinical trials.gov ID
ALS	ISIS 333611	2’-MOE	*SOD1*	SOD1^G93A^ transgenic rats Rhesus macaques	ICV	Discontinued	Ionis	NCT01041222
	Tofersen (BIIB067, ISIS 666853)	2’-MOE	*SOD1*	SOD1^G93A^ transgenic mice and rats Cynomolgus monkeys	ICV IT	Marketed	Ionis/Bioge	NCT02623699 NCT03070119
	BIIB078 (IONIS-C9)	2’-MOE	*C9ORF72*	*C9ORF72* BAC transgenic mice	ICV	Discontinued	Ionis/Biogen	NCT03626012
	Afinersen	2’-MOE/ PS	*C9ORF72*	*C9ORF72* BAC transgenic mice Sheep Cynomolgus monkeys	ICV IT	Phase I trial	RNA Therapeutics Institute at UMass Chan Medical School	IND141673
	WVE-004	Stereopure PN	*C9ORF72*			Discontinued	Wave Life Sciences	NCT04931862
	ION363 (Jacifusen)	N/A	*FUS*	FUS^P525L^ mice	ICV	Phase III	Ionis	NCT04768972
	BIIB105 (ION541)	2’-MOE	*ATXN2*	TDP-43 ALS mice	ICV	Discontinued	Biogen/Ionis	NCT04494256
HD	Tominersen (IONIS-HTT_Rx_, RG6042)	2’-MOE	*HTT*	Rhesus macaques	IT	Discontinued	Ionis/Roche	NCT03342053 NCT03761849 NCT02519036
	WVE-120101	2’-OMe/ stereopure PS	*mHTT*	BACHD mice YAC128 mice R6/2 mice	ICV (mice) IT (Rhesus macaques)	Discontinued	Wave Life Sciences	NCT03225833
	WVE-120102	2’-OMe/ stereopure PS	*mHTT*			Discontinued	Wave Life Sciences	NCT03225846
	WVE-003	2’-MOE	*HTT*			Phase Ib/IIa	Wave Life Sciences	NCT05032196

## Limitations of ASO therapy

### Blood–brain barrier (BBB) crossing

The high specificity and precise treatment of ASOs make them ideal candidates for the treatment of neurodegenerative diseases. However, the BBB and the blood-CSF barrier limit the passage of molecules, typically allowing passage of only hydrophobic small molecules with a molecular weight < 450 Da [107, 108]. Therefore, an effective method to deliver ASOs from the bloodstream to the brain would be highly beneficial. Early ASO delivery methods involve the use of viral vectors that carry cDNA in the antisense direction, which is transduced into the cell nucleus, and the resulting mRNA transcripts bind to the target sequence to silence the translation process. However, the size of the mRNA is a major drawback of this method and may lead to off-target effects [109]. Nanoparticles can be loaded with drugs through covalent or non-covalent interactions (such as hydrophobic, electrostatic and hydrogen bonding). Oligonucleotides carried by nanoparticles can easily traverse cell membranes, have a lower risk of being cleaved by nucleases, and exhibit higher bioavailability [110]. In 2021, Yokota et al*.* developed cholesterol-modified DNA/RNA hybrid double-stranded oligonucleotides (Chlo-HDOs), which can inhibit the expression of target genes in the brain and spinal cord through subcutaneous or intravenous injection. Chlo-HDOs can cross the BBB more efficiently without the need for CSF administration [111]. However, clinical trials are still needed to validate their efficacy and safety. Apolipoprotein A-I (apoA-I) is the major protein component of high-density lipoprotein (HDL) and plays a key role in cholesterol metabolism. HDL is involved in reverse cholesterol transport from peripheral tissues to the liver for excretion or redistribution to other tissues [112]. Hayden et al*.* utilized apoA-I ND (nanodisks) as a biocompatible carrier to enhance the delivery of ASOs targeting mHTT, into the brain and peripheral organs for the treatment of HD. When delivered intravenously in an HD mouse model, the ASOs significantly reduced mHTT in the liver, skeletal muscle, heart, and brain [113]. However, clinical trials are still needed to validate their efficacy and safety.

### Safety of ASO drugs

So far, most ASO drugs have been used to treat rare diseases for which there are no other treatment options, which means that the consideration of safety risks of the drugs has been somewhat outweighed by their therapeutic benefits. Like all drugs, ASOs exhibit dose-dependent toxicity. ASO toxicity includes (1) type I hypersensitivity reactions occurring after ASO injection, (2) off-target mRNA binding, which may reduce the levels of off-target proteins or alter their structure to impair function, and (3) chemical modifications that result in protein interactions with ASOs, leading to subsequent immune reactions and non-specific adverse events, including fever, thrombocytopenia, nephrotoxicity, and hepatotoxicity [114]. By analyzing adverse reactions in nonclinical studies and clinical trials, the nonclinical toxicity of ASO drugs is mainly characterized by immune reactions, thrombocytopenia, and toxicity to high-exposure organs (liver and kidney) [114, 115]. For example, in clinical trials, three patients treated with inotersen also experienced severe thrombocytopenia [116]. Although there is some understanding and speculation about the hepatotoxicity, nephrotoxicity, and hypersensitivity reactions caused by ASO drugs, the precise molecular and biochemical events underlying the toxic lesions at the genetic level are still unclear. It is also unclear which pharmacological mechanisms can mitigate these risks.

### High cost of ASO drugs

Nusinersen (ISIS-396443), which is already in the market, acts by binding to the splice site of exon 7 in *SMN2*, thereby reducing its truncation during splicing and increasing the production of full-length SMN protein. It is also the first drug approved by FDA for the treatment of SMA [117, 118]. However, the nusinersen (marketed as spinraza) has a high cost, with the first year of treatment priced at $750,000 (a single injection costs $125,000, with six injections per year), and subsequently reducing to $375,000 per year. The current high price is unaffordable for any healthcare system. With the expected entry of more ASO drugs into the market, healthcare companies, policymakers, and pharmaceutical companies urgently need to find ways to lower the pricing of ASO drugs [119].

### ASO drugs in large animal models

Most of the preclinical studies of ASO drugs were conducted in rodent models. However, studies of ASO drugs in large animal models began from 2009. In a study using the beagle dog model, ASO drugs were employed to treat DMD, marking the first report of ASO drugs rescuing dystrophin in a DMD dog model. The authors administered a combination of three equimolar ASO drugs via muscle injection and systemic intravenous infusion, with cumulative (combination) doses ranging from 120 to 200 mg/kg per injection. Systemic intravenous infusions were performed weekly or every two weeks, for 5 to 22 weeks. The experimental results demonstrated induction of therapeutic levels of systemic dystrophin expression, reduced inflammation observed through magnetic resonance imaging and histological examination, and no toxicity detected in blood tests [120]. In 2013, Kordasiewicz et al*.* further investigated intrathecal delivery of ASOs complementary to human *HTT* mRNA in the non-human primates (rhesus monkeys), continuously infusing a dose of 4 mg/day into the CSF for 21 days. Data showed ASO distribution in most cortical regions and pyramidal neurons, as well as a significant reduction of HTT protein to 47% of normal level in the prefrontal and occipital cortices [106]. Preclinical trials of ASOs in various animal models have shown minimal toxicity. While the ASOs demonstrate positive therapeutic effects in preclinical large animal models, challenges arise due to species differences and the complex etiology of diseases.

## Conclusions and future directions

Nucleic acid drugs including the ASOs offer a broader range of therapeutic applications compared to the small-molecule drugs. They have simpler designs that do not require consideration of protein three-dimensional structures, leading to shorter development cycles and faster drug target screening. Additionally, resistance to nucleic acid drugs is less likely to develop, and their effects are often long-lasting. With advancements in medical technology worldwide, small nucleic acid drugs like ASOs hold great potential for treating a wide range of neurodegenerative diseases, especially in conditions like ALS and HD. These drugs could revolutionize the treatment landscape for these diseases by offering targeted and potentially more effective therapeutic options.

## Data Availability

Not applicable.

## Abbreviations

ASO: Antisense oligonucleotide
ALS: Amyotrophic lateral sclerosis
ALSFRS-R: ALS functional rating scale-revised
ApoA-I: Apolipoprotein A-I
C9ORF72: Chromosome 9 open reading frame 72
CSF: Cerebrospinal fluid
CMV: Cytomegalovirus
CNS: Central nervous system
Chlo-HDO: Cholesterol conjugated heteroduplex oligonucleotide
DMD: Duchenne Muscular Dystrophy
FUS: Fused in sarcoma
FTD: Frontotemporal dementia
GOF: Gain-of-function
HD: Huntington’s disease
HTT: Huntingtin
HDL: High-density lipoprotein
IT: Intrathecal injection
ICV: Intra-cerebroventricular injection
MOE: Methoxyethyl
NfL: Neurofilament light chain
PNA: Peptide nucleic acid
PS: Phosphorothioate
PMO: Phosphorodiamidate morpholino oligomers
RNase H1: Ribonuclease H1
SOD1: Superoxide dismutase 1
SMA: Spinal muscular atrophy
SMN2: Survival motor neuron 2
SNP: Single nucleotide polymorphism
TARDBP: Transactive response DNA binding protein
UTR: Untranslated region
VMAT2: Vesicular monoamine transporter 2
